# Significant Stability Improvement of Fullerene Organic Photovoltaics via ZnO Film Modification through the Intermittent Spray Pyrolysis Technique

**DOI:** 10.1021/acsaem.1c03994

**Published:** 2022-03-29

**Authors:** Enas Moustafa, Lluis F. Marsal, Josep Pallarès

**Affiliations:** Department of Electrical Electronic Engineering and Automatic, Universitat Rovira i Virgili, 43007 Tarragona, Spain

**Keywords:** stability of fullerene organic photovoltaics, ZnO electron transporting layer, thin film deposition techniques, intermittent spray pyrolysis, degradation mechanisms in organic photovoltaics

## Abstract

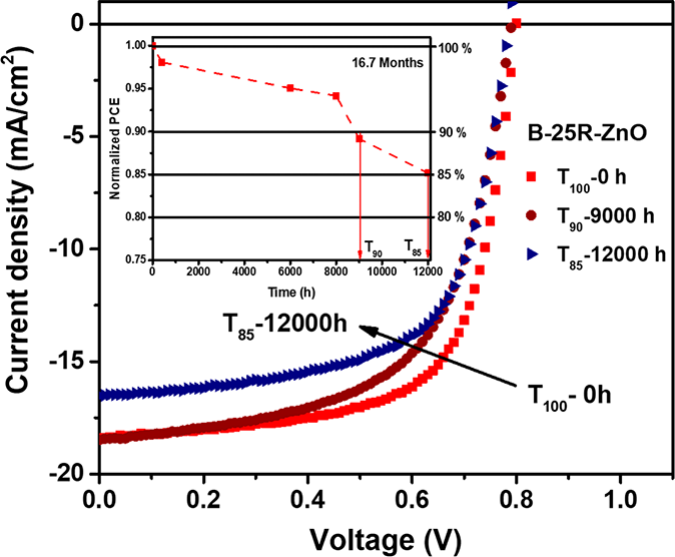

Morphological control of the layers within the bulk heterojunction organic photovoltaics (BHJ-OPVs) is a key feature that governs their performance. In the present work, we demonstrate that zinc oxide—ZnO—interlayers sprayed via the intermittent spray pyrolysis technique, employing a low-concentration precursor solution, can yield inverted BHJ-OPVs as efficient as the standard reported ones using the conventional laboratory-scale spin-coating technique. However, we record a pioneer stability behavior of the fabricated inverted fullerene organic photovoltaics (iF-OPVs) with various sprayed ZnO conditions. Thus, after optimizing the sprayed ZnO interfacial layer morphology for the inverted PTB7-Th:PC_70_BM devices, by carefully inspecting the interdependence between the sprayed ZnO thin film morphology and the figures of merit of the optimized iF-OPVs, we conducted a distinct analysis on the optical and electronic properties of the fresh and degraded devices using external quantum efficiency measurements and impedance spectroscopy. Hence, we showed that the most proper ZnO microstructural morphology was obtained by spraying 25 running cycles (25R). Remarkably, we observed that 25R-ZnO-based iF-OPV devices showed a stunning stability behavior and maintained 85% of their initial power conversion efficiency even after 16.7 months without encapsulation in a dry nitrogen glovebox, demonstrating an excellent shelf stability. Accordingly, this approach might facilitate the scalability of inverted OPVs for industrial production visibility.

## 1 Introduction

Since 2005, it has been proposed that polymer-based solution-processed organic photovoltaics (OPVs) with a 10% power conversion efficiency (PCE) would be adequate for initiating the commercialization, which would in turn assist the cost savings of broad materials development.^1^ However, production of OPVs on a large scale and at a competitive price is a breakthrough waiting to be achieved. Importantly, while OPVs will essentially continue incremental progress in the PCE, the devices will also require advanced stability for long-term operation to be able to compete in the commercial applications field. There are tremendous attempts that have been performed to understand the degradation mechanisms and improve the OPV cell stability, including the enhancement of the active blend materials, which indicated more photochemical stability,^2^ the insertion of the polymeric side chains that improve thermal stability,^3,4^ and the encapsulation techniques.^5,6^ Moreover, some other avenues involve interlayer morphology tuning to provide a better film quality through the selection of interfacial materials, interfacial device engineering, and the approach of the inverted structure along with the layer deposition techniques.^7−11^ Regarding the latest mentioned approaches, as shown by Lloyd et al.,^12^ MacLeod et al.,^13^ and several other reported studies, the inverted architectures has noticeably improved the OPV device stability over the conventional structures from the scale of minutes up to years.^13−15^ Therefore, with this context, we fabricated an inverted fullerene OPV (iF-OPV) based on selecting zinc oxide (ZnO) as the electron interfacial transporting material [electron transport layer (ETL)] for reinforcing the electron collection at the transparent conducting electrode and acquiring a high-performance and stable OPV.^13^ This selection was mainly regarding its n-type conductivity and high optical transmission at vital wavelengths for solar energy conversion applications.^16−19^ Furthermore, the ZnO thin film was subjected to various deposition techniques, including spin coating,^13,20^ atomic layer deposition,^21^ inkjet printing,^22−24^ and spray pyrolysis techniques.^7,8^ This in turn influences the morphology, substrate coverage, and contact quality (roughness) of the ZnO layer at the interface with the organic absorber blend. These parameters are the most noteworthy factors in identifying the performance and stability of OPV devices.^14,25^

In this piece of work, we report the use of an intermittent spray pyrolysis technique to modify and optimize the ZnO film using a low-concentration ZnO precursor solution as an extension of the previous work.^8^ Moreover, to focus on the study of the intrinsic degradation behavior of the fabricated devices, we stored and analyzed the samples in an inert atmosphere of <0.1 ppm O_2_ and <0.1 ppm H_2_O to avoid the possible degradations that might arise from the oxygen and the ambient moisture. Subsequently, we related the exhibited microstructural morphology of the ZnO film with the photovoltaic performance of the fabricated fresh and aged devices. The demonstrated PCE values of the fabricated devices are either better than or comparable to those in the previous reports obtained using different ZnO precursor solution concentrations as well as the conventional spin coating technique.^8,14,15^ The insights gained into the significance of the morphology of sprayed ZnO films highly assisted the development of the film quality.^13,19,25^ Hence, in this work, a novel and pioneer record behavior of the iF-OPVs is presented via a significant enhancement of its stability, achieving 12,000 h (16.7 months). This improvement is provided through the modification of the ZnO interfacial layer concentration along with the use of the spraying deposition parameters. Therefore, a systematic study was accomplished to reveal the dependance of the ZnO film morphology on the deposition parameters. Moreover, a distinct investigation was carried out on the electric properties and the corresponding charge transfer, separation, recombination mechanisms, and trap density through the impedance spectroscopy (IS) measurements of the fresh and degraded devices.

## 2 Experimental Methods

**Figure 1:**
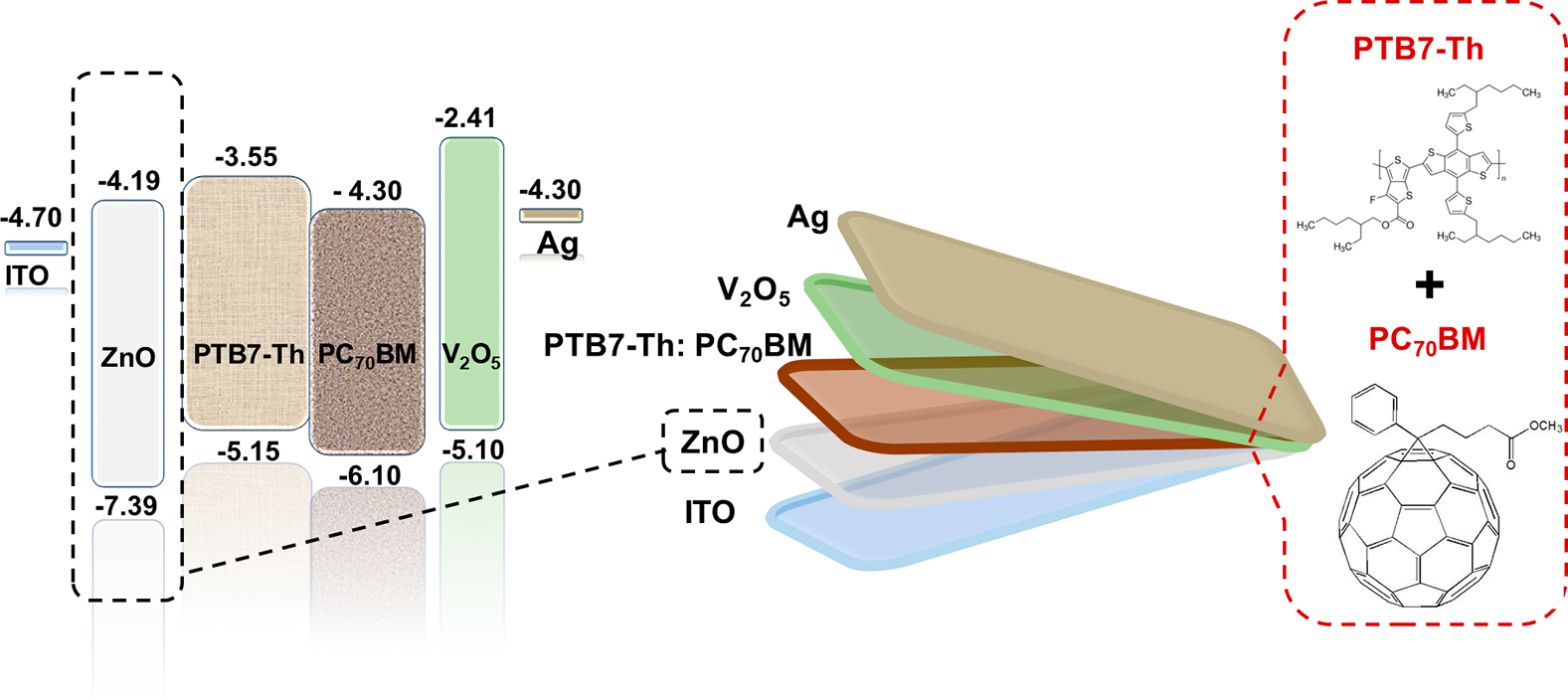
Scheme of the fabricated iF-OPV with the chemical structure of the PTB7-Th:PC_70_BM active blend and the energy level alignment of each layer within the fabricated device.

This section reports the materials, synthesis, and characteristics of the iF-OPVs with an ITO/ZnO/PTB7:Th:C_70_BM/V_2_O_5_/Ag structure, as demonstrated in Figure 1.

### 2.1 Materials

The transparent conducting oxide used was an indium tin oxide (ITO) patterned glass substrate with a sheet resistance of 10 Ω·cm^–2^ purchased from PsioTec Ltd. Donor polymer PTB7:Th and fullerene acceptor PC_70_BM were supplied by One-Material Inc. and Solenne BV, respectively. Zinc acetate dehydrate (99.999%), 2-methoxyethanol (99.9%), ethanolamine (99.5%), methanol, ethanol, chlorobenzene (CB), and 1,8-diiodooctane (DIO) were obtained from Sigma-Aldrich. Vanadium oxide (V_2_O_5_) and a high-purity silver (Ag) wire (99.99%) were purchased from Sigma-Aldrich and Testbourne Ltd., respectively.

### 2.2 Device Fabrication

Prepatterned ITO-coated glass substrates were subsequently cleaned with a detergent in deionized water and then ultrasonicated in acetone, ethanol, and isopropyl alcohol. Then, the ITO substrates were dried in a 100 °C oven for 10 min and exposed to UV–ozone for 15 min. The ZnO precursor solution was synthesized using the sol–gel method according to the previously reported studies of our group^8,22,26,27^ by dissolving 150 mg of zinc acetate dihydrate in 1 mL of 2-methoxyethanol and 30 μL of ethanolamine solution. The prepared mixture was stirred at 70 °C for 1 h and then diluted to a 1:1 (v/v) ratio of methanol to prepare the ZnO precursor stock solution. This solution was further diluted by ethanol with a 0.5:9.5 (v/v) ratio and then sprayed over the preheated ITOs at 350 °C. Then, the ZnO sprayed films were annealed for 1 h and removed to cool down. The used spray pyrolysis tool along with the procedure details used to spray the ZnO solution using the intermittent spray pyrolysis approach was described in our previously reported work.^8^ In the current work, various numbers of spraying running cycles (R) of 15R, 20R, 25R, and 30R were performed to deposit different thicknesses of the ZnO film. Each intermittently spraying running cycle (1R) step is defined as 7 s of continuous spraying and 3 s of hold, followed by repeating the same attitude till the corresponding number of spraying running cycles required has been sprayed.

The polymer blend solution was synthesized by dissolving 25 mg of PTB7-Th:PC_70_BM in 1 mL of CB/DIO (97:3, % v/v) at a 1:1.5 (w/w) ratio. Before deposition, the solution was retained under stirring at 40 °C for 48 h. An approximately 100 nm film of the PTB7-Th:PC_70_BM active blend was spin-coated (750 rpm, 30 s) on glass/ITO/ZnO inside the glovebox.^8,15,22^ Finally, the samples were conducted in a vacuum chamber to evaporate V_2_O_5_, followed by Ag films under high-vacuum conditions (≤1 × 10^–6^ mbar) to acquire thicknesses of 4 and 100 nm, respectively. To ensure the obtained cell area, we used a shadow mask with an area of 0.09 cm^2^ during the previously mentioned thermal evaporation process.

### 2.3 Device Measurement and Characterization

The current density–voltage (*J*–*V*) characteristics parameters were extracted using a Keithley 2400 source measure unit with a solar simulator light source (Abet Technologies model 11,000 class type A). The AM 1.5G (100 mW cm^–2^) standard light intensity of the spectrum was calibrated using a certified monocrystalline silicon photodiode of NREL. The *J*–*V* measurements were conducted in the dark to obtain *J*–*V* dark curves. *J*–*V* curves were obtained from −1 to 1 V in the forward direction, with a scan step of 0.01 V. All the *J*–*V* measurements were conducted at room temperature. Moreover, the external quantum efficiency (EQE) measurement was performed using a Lasing IPCE-DC model under a forward wavelength sweep direction from 300 to 800 nm. In addition, IS measurements were achieved using an HP-4192A analyzer under an AM 1.5G illumination condition. Impedance data were acquired in the frequency range between 5 Hz and 1 MHz by applying an AC signal with a 5 mV amplitude. The devices were measured in a carefully sealed holder inside the glovebox. IS experimental data were fitted using an Ivium software analyzer. It is worth noting that the device performance was measured both prior to and after the impedance measurements, presenting no significant degradation. ZnO film topography images were derived via a tapping mode of atomic force microscopy (AFM) using silicon probes of 1–5 N m^–1^ spring constant. Furthermore, a Molecular Imaging Pico SPM II instrument (pico+ software) was used to analyze the ZnO surface morphology images, providing the root mean square (rms) values. The film thickness within the devices was measured using the surface profilometer (Ambios Technology-XP 1). Finally, the transmittance spectra were measured using a PerkinElmer LAMBDA 950-UV/vis/NIR spectrometer integrating sphere at room temperature.

## 3 Results and Discussion

An intermittent spray pyrolysis approach^8^ has been used to optimize the deposition of the ZnO film through different numbers of running cycles (R) of the ZnO precursor solution (as described in the Experimental Methods section). We focus our investigation toward modifying the substrate coverage and surface morphology of the ZnO films and pointing out the morphological compact spot where ZnO films can achieve a similar benchmark efficiency along with an improved stability behavior. The influence of these optimization procedures on the performance and stability of iF-OPVs was studied by fabricating the inverted structure devices of ITO/ZnO/PTB7-Th:PC_70_BM/V_2_O_5_/Ag, as shown in Figure 1. ZnO was used as the n-type charge transport layer and V_2_O_5_ was used as the p-type charge transport layer. Furthermore, PTB7-Th:PC_70_BM was employed as the photo-absorber active layer. The chemical structures of the PTB7-Th polymer donor and PC_70_BM fullerene acceptor with the energy level alignment of the devices are illustrated in Figure 1. The semiconductor band edge energy positions and the metal work functions were provided from refs (15), (20), (28), and (29).

**Figure 2:**
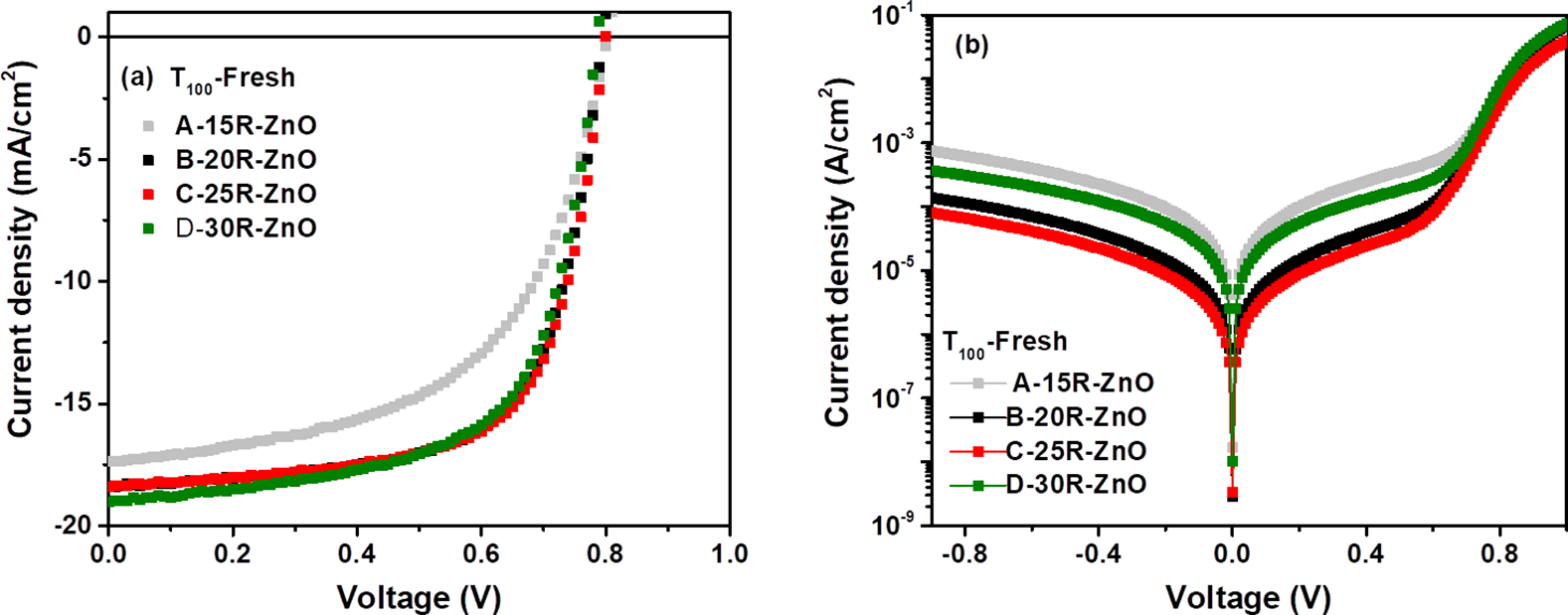
Current density–voltage (*J*–*V*) characteristic curves (a) under AM 1.5G illumination and (b) under a dark condition for fresh iF-OPVs.

To easily distinguish the optimization performed on the ZnO film, we labeled the fabricated iF-OPVs as A, B, C, and D for the devices with 15R, 20R, 25R, and 30R as the number of r spraying running cycles to prepare the ZnO layer, respectively. Figure 2 displays the current density–voltage (*J*–*V*) characteristics of the freshly fabricated iF-OPVs (*T*_100_—the time of the initial PCE, fresh devices) with different processing methods of ZnO spraying running cycles measured under AM 1.5G (100 mW/cm^2^) and dark conditions. The obtained photovoltaic parameter statistics of the corresponding devices are listed in Table 1. As demonstrated in Figure 2a, it can be noticed that all the devices have almost the same open-circuit voltage (*V*_OC_) value. Moreover, C-based devices (25R-ZnO) demonstrated the champion performance as it exhibited the maximum PCE of 9.86%, with an average *V*_OC_ of 0.8 V, a current density (*J*_SC_) of 18.42 mA cm^–2^, a fill factor (FF) of 0.67, a series resistance (*R*_S_) of 2.31 Ω cm^2^, and a shunt resistance (*R*_Sh_) of 774 Ω cm^2^. On the other hand, device A (15R-ZnO) possessed the lowest performance parameters with the maximum PCE, average *V*_OC_, *J*_SC_, FF, *R*_S_, and *R*_Sh_ of 7.76%, 0.78 V, 17.17 mA cm^–2^, 0.54, 4.63 Ω cm^2^, and 429 Ω cm^2^, respectively. In addition, device B (20R-ZnO) showed performance parameters with the maximum PCE, average *V*_OC_, *J*_SC_, FF, *R*_S_, and *R*_Sh_ of 9.80%, 0.79 V, 18.26 mA cm^–2^, 0.66, 2.83 Ω cm^2^, and 765 Ω cm^2^, respectively, and device D (30R-ZnO) with 9.60%, 0.76 V, 18.88 mA cm^–2^, 0.63, 3.07 Ω cm^2^, and 381 Ω cm^2^, respectively. It is worth mentioning that the number of spraying running cycles (R) controls the thickness of the formed ZnO film. Hence, we observed that as the ZnO thickness increases (R-increases), the *J*_SC_ of the fabricated devices improves. However, in device D (30R-ZnO), the slight enhancement of the *J*_SC_ was at the expense of a marginal reduction of the *V*_OC_ and FF, which as a consequence diminishes the PCE as shown in Table 1. Figure 2b shows the dark *J*–*V* curves for the freshly fabricated cells. Under a reverse bias, we obtained a 1 order of magnitude lower leakage current for B and C devices than for A and D ones.

**Table 1:** Photovoltaic Performance Parameter Statistics of the Devices, Extracted from the Average of at Least Nine Devices

device *T*_100_	*V*_OC_ (V)	*J*_SC_ (mA/cm^2^)	FF	PCE (%)	PCE_MAX_ (%)	*R*_S_ (Ω cm^2^)	*R*_SH_ (Ω cm^2^)
A-15R-ZnO	0.78 ± 0.02	17.17 ± 0.20	0.54 ± 0.02	7.53 ± 0.23	7.76	4.63 ± 0.41	429 ± 26
B-20R-ZnO	0.79 ± 0.01	18.26 ± 0.12	0.66 ± 0.01	9.71 ± 0.09	9.80	2.83 ± 0.32	765 ± 17
C-25R-ZnO	0.80 ± 0.02	18.42 ± 0.19	0.67 ± 0.04	9.80 ± 0.11	9.86	2.31 ± 0.11	774 ± 24
D-30R-ZnO	0.78 ± 0.01	18.88 ± 0.31	0.63 ± 0.03	9.46 ± 0.14	9.60	3.07 ± 0.43	381 ± 19

This result of less leakage current in the B and C devices might be attributed to the higher shunt resistance *R*_SH_(30) values of 1.64 × 10^4^ in device B and 2.69 × 10^4^ Ω cm^2^ in device C than the values of 2.32 × 10^3^ of device A and 3.88 × 10^3^ Ω cm^2^ of device D in the dark. This enhancement in the *R*_SH_ in the B- and C-based devices discloses the lower charge carrier recombination in the active blend film.^31^ This behavior agrees with the enhanced FF observed for devices B and C (Table 1). In addition, the diminished FF and *J*_SC_ value for device A under illumination (Figure 2a) matches the highest leakage current obtained in the dark that is shown in Figure 2b.

**Figure 3:**
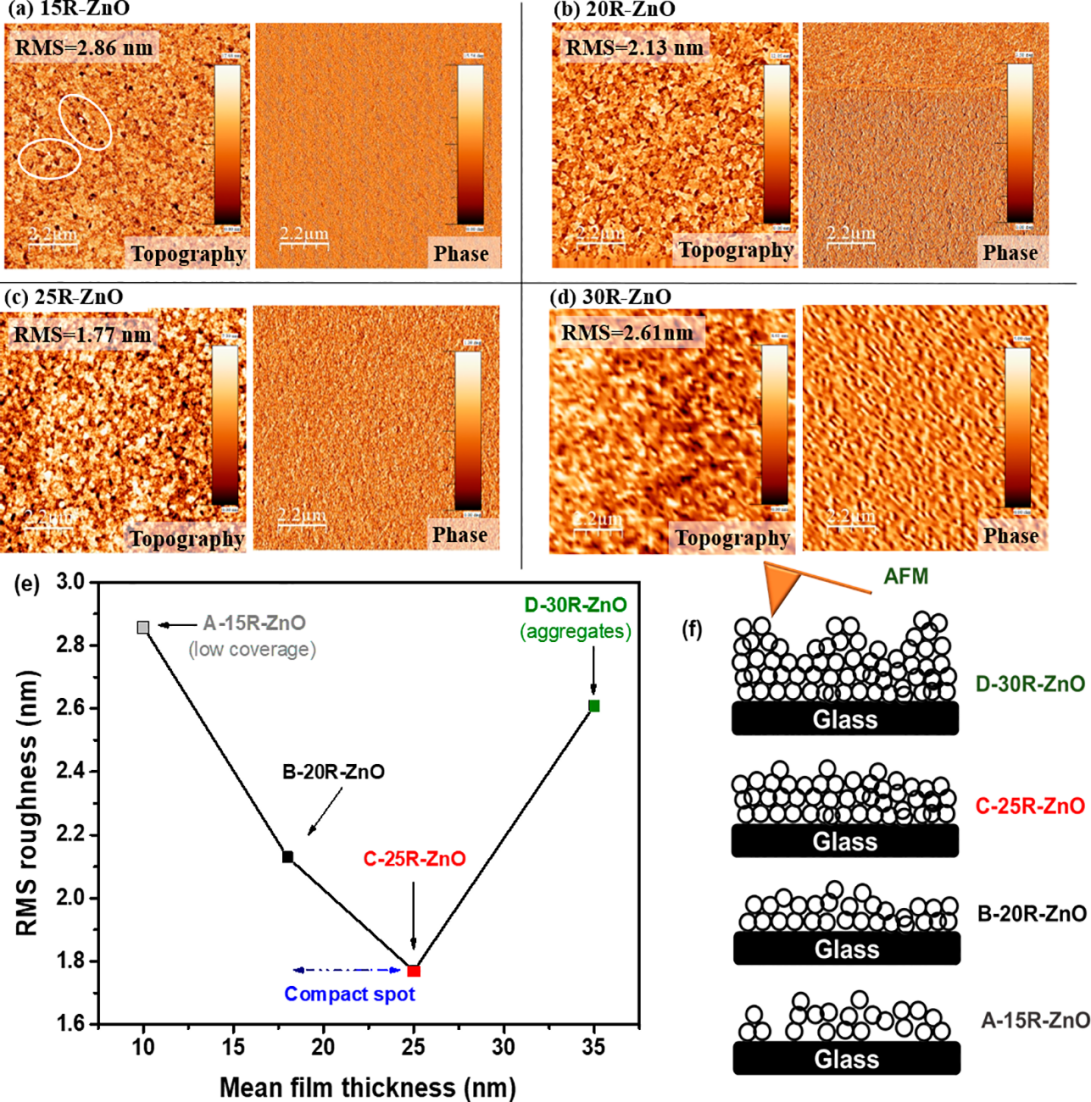
AFM topographic and phase images of the ZnO film with (a) 15R (thickness = 10 nm), (b) 20R (thickness = 18 nm), (c) 25R (thickness = 25 nm), and (d) 30R (thickness = 35 nm). (e) rms surface roughness of ZnO films as a function of film thickness. (f) Schematics simplifying the evaluated ZnO surface morphology measured using the AFM tip.

Figure 3 illustrates the AFM surface topographic and phase images of the ZnO thin film deposited via the spray pyrolysis technique onto the glass substrate to investigate the morphological variations of the sprayed ZnO films. The average thickness of the ZnO films was optimized as a function of the number of spraying running cycles, where 15R-ZnO, 20R-ZnO, 25R-ZnO, and 30R-ZnO with the varying number of running cycles correspond to 10, 18, 25, and 35 nm, respectively. As can be noticed in the 15R-ZnO film in Figure 3a, it contains many defects that lead to an erratic surface with an rms of 2.86 nm. Although after increasing the number of spraying running cycles to 20R and 25R, the thicknesses increased, the ZnO film obtained was featureless and homogeneous, which leads to a roughness reduction with rms values of 2.13 and 1.77 nm, respectively, as presented in Figure 3b,c. In addition, upon further increasing the spraying running cycles to 30R, the roughness increased to an rms of 2.61 nm (Figure 3d). Interestingly, the same behavior was obtained by Jagadamma et al. and Ma and co-workers.^14,25^ The relation between the ZnO film thicknesses and the rms values is demonstrated in Figure 3e. We imply that the effect might be due to the ZnO surface coverage during the ZnO precursor solution spraying processes, as simplified in Figure 3f, where in the 15R-based film, the surface was not fully covered, which caused some surface imperfection. This basically matches with the low FF and *R*_Sh_ values and high *R*_S_ values observed from the A-based cells. Then, by increasing the number of spraying running cycles to 20R and 25R, we noticed that the film roughness diminished, reflecting a uniform sprayed ZnO film along with better density and quality. This surprising behavior confirms the higher *R*_Sh_ and FF values and the lower *R*_S_ values obtained for B- and C-based devices. Moreover, regarding the 30R-ZnO film, the higher roughness might be attributed to the excess ZnO precursor solution sprayed that leads to some surface aggregations and a non-homogeneous ZnO film, which may cause the increase of the film roughness. Consequently, we observed that 15R- and 30R-based ZnO films possessed the highest surface roughness, which indicates the higher leakage current^32^ obtained from A- and D-based devices, as previously discussed in Figure 2b. It is worth noting that as compared to our previously reported work^8^ as well as the work of Jagadamma et al.,^14^ we found out that the mentioned surface modification performed for the ZnO film in the current work via the intermittent spray pyrolysis approach using low concentration of ZnO solution (Experimental Methods) suppressed the surface roughness of the deposited ZnO film, exhibiting better film quality along with providing higher device performance.

**Figure 4:**
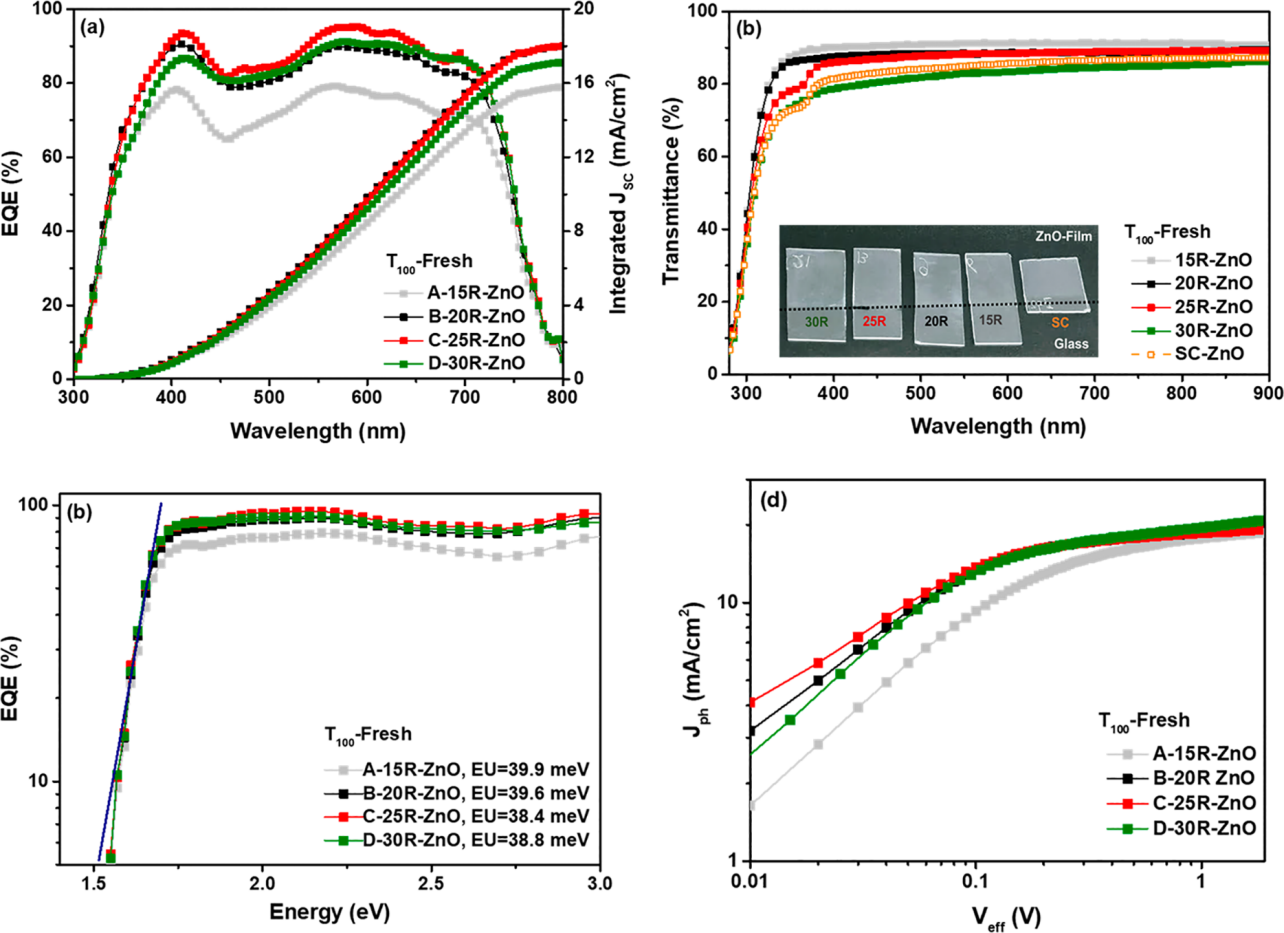
(a) EQE spectra (left) and the integrated short-circuit current (right) of the freshly fabricated iF-OPVs, (b) UV–vis optical transmittance characteristics of the various ZnO films deposited using intermittent spray pyrolysis (15R, 20R, 25R, and 30R) along with the ZnO film coated using the spin coating technique reported in our previous work.^8^ (c) EQE vs photon energy of the fresh iF-OPVs with the inset values of Urbach energy (*E*_U_), and (d) *J*_Ph_ vs *V*_eff_ curves of the fresh iF-OPVs with ZnO interfacial layers of various thicknesses.

Figure 4a shows the EQE response of the fabricated devices. As shown, the trend of the EQE is almost similar to that of *J*_SC_ of the fabricated iF-OPVs with ZnO films of different thicknesses. Regarding the absorption spectra of donor and acceptor materials, all devices display photo-response spectra with a broad range of 300–800 nm, which is mainly attributed to the absorption spectra of the PTB7-Th:PC_70_BM blend.^27^ Devices based on A-15R-ZnO showed the lowest maximum plateau photoresponse of 80%, while B- and D-based devices possessed 90% and C devices exhibited the highest plateau value of 95%. This observed reduction in the spectra of device A might be related to the insufficient charge extraction and transport mechanism that highly matches their low obtained performance parameters, as listed in Table 1. Furthermore, we note that the calculated integrated *J*_SC_ values from the EQE spectra are 15.79, 17.98, 18.01, and 17.12 mA cm^–2^ for A, B, C, and D devices, respectively. These calculated *J*_SC_ values basically confirm the obtained values by the *J*–*V* measurements under illumination with an approximate error percentage of less than 10 (Table 1).

To further understand the influence of different numbers of R used to fabricate the ZnO film on the underlying PTB7-Th:PC_70_BM blend films and investigate the relation regarding the enhancement of the *J*_SC_ in the fabricated cells, we measured the UV–visible transmittance spectrum of the sprayed ZnO films with different, R, thicknesses, as presented in Figure 4b. On one hand, we compare the effect of the deposition techniques on the transmittance (*T* %) of the fabricated ZnO film. As is seen in Figure 4b, we demonstrated the transmittance of ZnO films fabricated using the spin coating technique (ZnO-SC) in the previously reported work^8^ and the current studied ones deposited using the intermittent spray pyrolysis technique. It was interesting to manifest that the ZnO film sprayed using the spray pyrolysis technique (ZnO-SP) exhibited higher *T* % than the ZnO film deposited using the spin coating technique (ZnO-SC). This can be noticed by the naked eye, as demonstrated in the inset photos of the ZnO films in Figure 4b. This means that the ZnO-SP films allow more light to be passed to be absorbed by the active layer and therefore enhance the photogeneration of charges. Interestingly, this behavior was confirmed by the UV–visible absorbance spectra of the ZnO/active blend for each device configuration, as illustrated in Figure S1. This reveals the enhancement of the *J*_SC_ values that was noticeable for the ZnO-SP based devices. On the other hand, in the current work, we compare the transmittance of the ZnO sprayed films with different thicknesses. The obtained results followed the logical trend where transmittance decreases as the thickness increases. Furthermore, it is important to mention that the A-15R-ZnO-based film, however, has the lowest thickness, the highest transmittance (Figure 4b), and high absorbance respond (Figure S1), but it exhibited the lowest *J*_SC_ value (Table 1). This might be attributed to the major defects obtained in the 15R-ZnO-film, as discussed previously (Figure 3a), that led to the higher film roughness. This behavior devoted the high *R*_S_ and low *R*_Sh_ values that explain the diminishment in the FF and as a consequence the reduction of the PCE of A-based devices (15R-ZnO) (Table 1). Moreover, we can see that the D-30R-ZnO-film possessed the lower transmittance (Figure 4b), but the D-based devices showed the highest *J*_SC_ value, which might be attributed to the highest absorbance responses observed in Figure S1. A similar behavior was noticed in our previously reported work,^8^ which suggested it to be related to the film roughness. As the film roughness increases, it assists the light trapping inside the solar cells, which plays an important role in increasing the light absorbance by the active absorber layer.^33,34^ Consequently, higher photogeneration of charges can be exhibited, resulting in a higher *J*_SC_ value of the fabricated devices.

Furthermore, it was interesting to observe the reason behind the diminishment of the *J*_SC_, *V*_OC_, and PCE of the A-based devices by investigating the blend film optical properties using the Urbach equation given below^35,36^
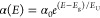
where α(*E*) is the optical absorption coefficient, α_0_ is the band edge optical absorption coefficient, *E* is the photon energy, and *E*_U_ is the Urbach energy. The *E*_U_ value describes the energetic disorder in the molecular orbitals as it features the density of states (DOS) distribution.^35^ Accordingly, the lower *E*_U_ value reveals the abrupt band edge.^26,36^Figure 4c shows the double logarithmic scale of the EQE versus the photon energy to manifest the effect of the ZnO film thickness sprayed using the spray pyrolysis technique on the optical properties of the blend active layer. We noticed that all the devices obtained a similar slope of tail-state distribution in the low-energy region. This behavior describes the quiet negligible effect of the energetic disorder of the active blend.^8^ Moreover, the calculated *E*_U_ values (in the inset of Figure 4c) were a bit close for all cells; however, the lowest *E*_U_ value was obtained for device C. This might reflect the less disorder distribution induced in the C-based devices that matches with their highest *V*_OC_ value and provides the champion performance among the other cells (Table 1). The same behavior can be noticed for the A-based devices, which have the highest *E*_U_ value. This indicates that a bit higher disorder suppresses the *V*_OC_, *J*_SC_, and FF of A-based devices and consequently the device PCE, as confirmed by the *J*–*V* characteristics (Figure 2 and Table 1).

It is known that the charge carrier extraction possibility can be used to unravel the mechanisms behind the current loss during charge extraction, which is basically calculated by the carrier transport layers and the interfaces between these layers and the active layers.^37,38^ Accordingly, for a more consistent comparison between the fabricated iF-OPVs, we calculated the dependence of the photocurrent (*J*_Ph_) on the effective voltage (*V*_eff_) to determine the exciton dissociation probabilities (*P*_diss_), the generation rate (*G*_rat_) of the free charge carriers, and the maximum amount of absorbed photons that impart the dissociation and generation of free carriers (*G*_max_) in the fabricated iF-OPVs.^39,40^*J*_Ph_ is described as *J*_L_ – *J*_D_, where *J*_L_ is the current density under light illumination and *J*_D_ is the current density in the dark. *V*_eff_ is defined as *V*_O_ – *V*, where *V*_O_ is the voltage when *J*_Ph_ = 0 and *V* is the applied voltage.^41−43^ The *G*_max,_ values were obtained by calculating *G*_max_ = *J*_sat_/*qL*,^39,40,43,44^ where *J*_sat_ is the saturation current density, *L* is the thickness of the blend film, and *q* is the elementary charge. Then, we evaluated *P*_diss_ and *G*_rat_ values from the equations^39,40,43,44^*P*_diss_ = *J*_SC_/*J*_sat_ and *G*_rat_ = *P*_diss_*G*_max_, respectively. Table 2 lists the calculated optoelectronic parameters from the *J*_ph_–*V*_eff_ characteristics.

**Table 2:** Optoelectronic Parameters Calculated from the *J*_ph_–*V*_eff_ Curves of the Freshly Fabricated iF-OPVs

device *T*_100_	*J*-sat (mA/cm^2^)	*G*_max_ (×10^28^ m^–3^ s^–1^)	*P*_diss_ (%)	*G*_rat_ (× 10^30^ m^–3^ s^–1^)
A-15R-ZnO	17.80	1.11	97.60	1.08
B-20R-ZnO	18.66	1.16	98.51	1.15
C-25R-ZnO	18.75	1.17	98.24	1.15
D-30R-ZnO	19.60	1.22	97.15	1.19

In Figure 4d, we depicted the curves of *J*_ph_–*V*_eff_ in a double logarithmic scale for the cells. The exhibited results denoted that the *J*_Ph_ of the devices increased linearly at low *V*_eff_ (*V*_eff_ < 0.5 V for A-based devices and *V*_eff_ < 0.2 V for the B-, C-, and D-based devices) and then it tends to saturate indicating the proper charge carrier separation.^39^ Moreover, the *J*_sat_ values evaluated from Figure 4d showed that B-, C-, and D-based cells have more efficient charge carrier separation within the interfaces of the active absorber layer^39,42^ than the A-based devices (Table 2). In addition, the values of the *G*_max_ for the A, B, C, and D devices were 1.11 × 10^28^, 1.16 × 10^28^, 1.17 × 10^28^, and 1.22 × 10^28^ m^–3^ s^–1^, respectively. It is interesting to notice that the highest value of the *G*_max_ was for D-based devices corresponds with its highest *J*_SC_ value obtained from the *J*–*V* characteristics under illumination (Figure 2a). In turn, this behavior matched with the *G*_rat_ values (Table 2) of the devices, indicating the effective dissociation of the photogenerated excitons for B-, C-, and D-based devices compared to that for A cells, which is consistent with the corresponding performances of the devices (Table 1). However, the *P*_diss_ value was higher for B and C devices than for A and D ones, which correlates with their highest FF and PCE values (Table1). In addition, the maximum value of *P*_diss_ was achieved for the C-based devices (98.51%) with a 25R-ZnO film of 25 nm thickness, which may imply that the 25R modification condition provided the highest level of surface passivation to the device.^37^ This observation is highly matched with the lowest film roughness obtained for the 25R-ZnO film showing better surface coverage, as shown in Figure 3.

In the following part, we performed a degradation study of the fabricated devices by storing them inside a dry glovebox under dark conditions in an electronic grade of 99.999% N_2_ (H_2_O humidity < 0.1 ppm and oxygen < 0.1 ppm) during the entire examination time. The samples are exposed to light during the electrical measurements and then stored back in the glovebox. The goal of this study is to manifest the modification effect of the ZnO film using the intermittent spray pyrolysis approach on the stability behavior of the fabricated devices. In addition, it is crucial to indicate the intrinsic degradation mechanisms upon the influence of the interfacial layers.

**Figure 5:**
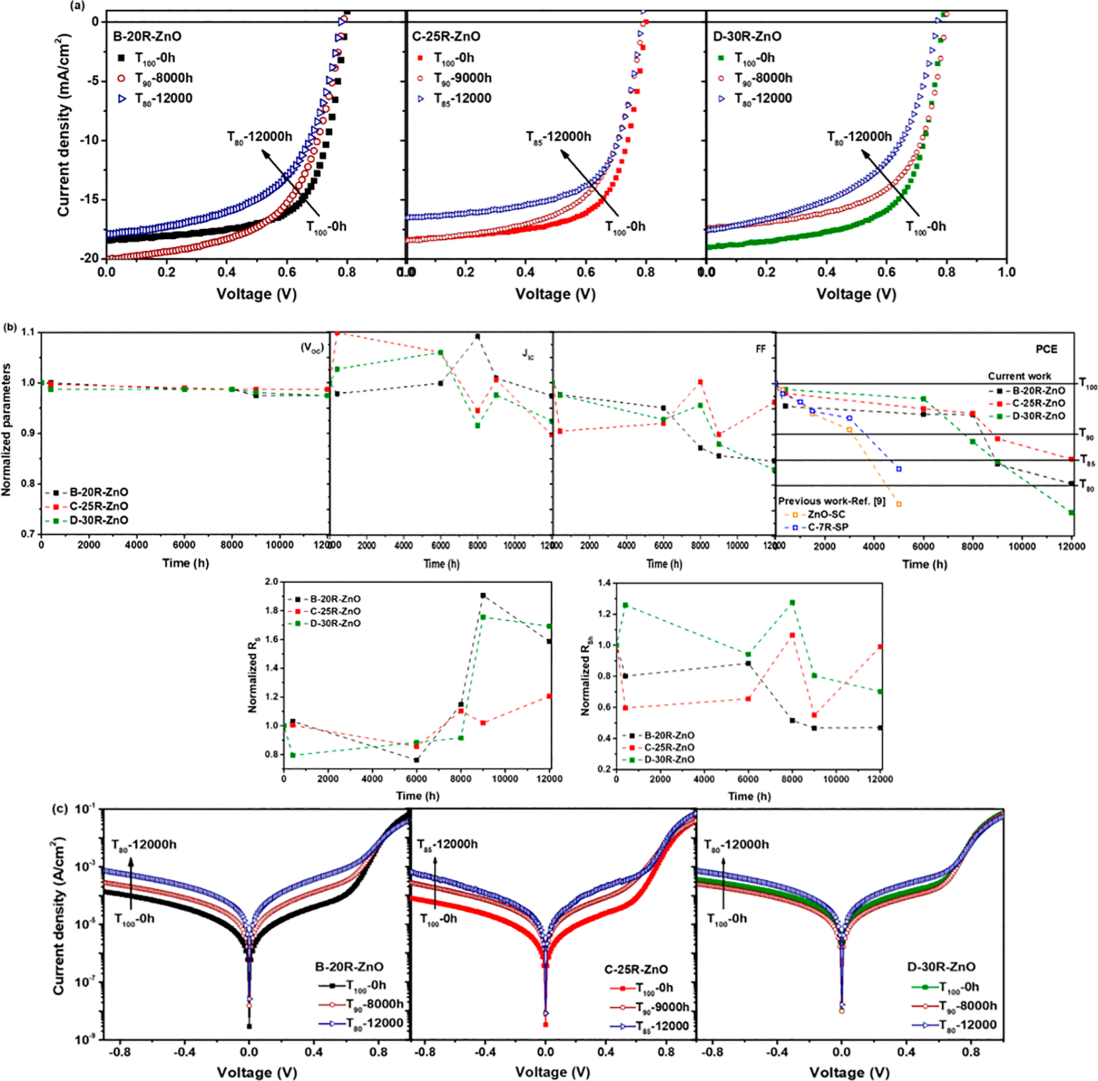
(a) Current density–voltage (*J*–*V*) characteristic curves under AM 1.5G illumination, (b) normalized performance parameters, and (c) *J*–*V* characteristic curves in the dark of the degraded iF-OPVs with respect to the aging time (12,000 h)—device stability study.

Figure 5a shows the illuminated *J*–*V* characteristics of the studied devices with respect to the aging time until they achieved about 80% of their initial efficiencies, known as *T*_80_. The *T*_100_ (the time of the initial PCE), *T*_95_ (the time for reaching 95% of the initial efficiency value), *T*_90_ (the time for reaching 90% of the initial efficiency value), and *T*_85_ (the time for reaching 85% of the initial efficiency value) are described in the protocol.^45^ More detailed photovoltaic performance statistics of the devices over time are summarized in Table S1. In addition, the decrease in the performance of the degraded devices over time is described in Figure S2.

It is worth mentioning that A-15R-ZnO-based devices have been excluded from the degradation comparison as it showed the lowest performance behavior. Hence, we focused on comparing the champion devices of C-25R-ZnO with the high-performance iF-OPVs of B-20R-ZnO and D-30R-ZnO ones. On one hand, it can be generally observed that the *J*–*V* curves of the entire degraded cells (after 12,000 h) showed a pronounced shift to lower *J*_SC_ with respect to the fresh devices (*T*_100_). On the other hand, the change in the *V*_OC_ was not noticeable for the degraded cells compared to that for the fresh ones. These behaviors are clearly observed in Figure 5b for the normalized *V*_OC_ and *J*_SC_ parameters of the degraded devices over aging time.

Moreover, it was interesting to notice that devices B and D reached 80% of their initial PCE after 12,000 h (16.7 months), while devices C still retained 85% of their initial PCE, as illustrated in the plot of the normalized PCE with respect to the time in Figure 5b. Remarkably, by considering our previously reported work,^8^ we can clearly notice that the ZnO-SC and C-ZnO-SP (sprayed via 7R high concentration of the ZnO precursor solution) iF-OPV devices achieved almost *T*_80_ after 5000 h only, demonstrating lower stability performance than the current work, as illustrated in Figure 5b.

Furthermore, the FF showed a more stable behavior for C-based devices than B and D cells, where after 12,000 h, the FF values were 0.66, 0.57, and 0.57 for C, B, and D devices, respectively (Table S1). This might be attributed to the slight increase as well as the marginal decrease of the *R*_S_ and *R*_Sh_, respectively, of C-based devices during the degradation time, as presented in Figure 5b. Accordingly, it might be the main reason behind devices C retaining the highest performance after 12,000 h, rendering the most stable behavior among the other devices.

It was interesting to observe an increase in the *J*_SC_ values of the B-20R-ZnO device after 8000 h and then a decrease again till *T*_80_. A similar behavior was exhibited for the C-25R-ZnO and D-30R-ZnO devices after 9000 h, followed by a decrease till 12,000 h. This increment in the *J*_SC_ values may originate from the UV irradiation dependence, reported as the light-soaking effect occurred during the measurements of the degraded devices, when the UV components from the solar simulator spectrum irradiate the cells.^46^ This phenomenon was widely observed for the degraded inverted OPVs using ZnO or other metal oxide materials as the ETL.^46^ There are two main suggested mechanisms behind the origin of this phenomenon. First, it may originate from the photo-induced rearrangement of the Fermi levels at the ITO/metal oxide interfaces upon the filling of trap states during the light exposure, which diminishes the potential barrier and thus enhances the electron extraction through the ITO/metal oxide interface.^46^ Second, the interfacial dipole clue role between the metal-oxide and the organic blend film interface as reported elsewhere.^47^ This behavior was confirmed by the small reduction in the *R*_S_ values during degradation (Figure 5b), followed by a rapid increase, which reflects the slightly enhanced FF as well, and then a decrease again till 12,000 h for the degraded devices (Figure 5b).

Hence, considering these obtained results along with the compared Jagadamma et al. reported work^14^ carried out with the same condition of degradation providing a maximum of 13 months of degradation, we indicated a higher performance as well as stability behavior for the C-based device regarding the modified ZnO film sprayed with 25R. In addition, based on the context in Table 3, it is worth clarifying that our modified ZnO film-based iF-OPV devices presented a pioneer record of stability behavior among the previously reported studies using the same photoactive absorber blend in a N_2_ atmosphere without encapsulation.

**Table 3:** Photovoltaic Performance Parameters of the Previously Reported iF-OPV Devices before and after Aging; PCE_A_: the PCE of the Aged Devices and PCE_F_: the PCE of the Fresh Devices

device structure	evaluated aging time (h)	*V*_OC_ (V)	*J*_SC_ (mA/cm^2^)	FF	fresh PCE (%)	aged PCE (%)	PCE_A_/PC*E*_F_ (%)	refs
C-25R-ZnOITO/ZnO/Blend/V_2_O_5_/Ag	12,000	0.80	18.42	0.67	9.86%	8.40	85	this work
ZnO-SCITO/ZnO/Blend/MoO_*X*_/Ag	8600	0.74	14.20	0.63	6.50	5.85	90	(14)
ZnO-SCITO/ZnO/Blend/V_2_O_5_/Ag	5000	0.79	17.69	0.73	10.19	7.77	76	(8), previous work
C-7R-ZnO-SPITO/ZnO/Blend/V_2_O_5_/Ag		0.79	18.46	0.68	10.00	8.32	83	
ITO/PEDOT:PSS/Blend/PFN/Ag	2400^a^	0.74	19.30	0.57	8.20	4.10	50^a^	(49, 50)
Inv-ML-STITO/ZnO/Blend/MoO_*x*_/Ag	1900	0.75	10.0	0.72	5.37	5.14	80	(51)
Inv-STITO/ZnO/Blend/MoO_*x*_/Ag	750	0.73	8.09	0.74	4.39	3.51		
Inv-OpaqueITO/ZnO/Blend/MoO_x_/Ag	250	0.75	13.26	0.73	7.27	5.82		
ITO/ZnO/Blend/MoO_*x*_/Ag	1000	0.82	14.19	0.53	6.25	3.83	61	(52)
ITO/PFN/Blend/MoO_*x*_/Ag	1460	0.81	16.2	0.68	8.9	4.81	54	(53)
ITO/FGr/Blend/MoO_*x*_/Ag		0.81	17.2	0.68	9.5	9.03	95	
ITO/PEDOT:PSS/Blend/PDINO/Ag	630	0.79	16.22	0.68	8.78	4.66	53	(54)
ITO/BiOCl-NPs/Blend/PDINO/Ag		0.79	18.42	0.68	9.92	7.92	80	
ITO/TiO_*X*_/Blend/MoO_*x*_/Ag	500	0.71	14.80	0.62	6.5	2.28	35	
ITO/TiO_*X*_/TPPZn/Blend/MoO_*x*_/Ag		0.72	14.90	0.66	7.1	3.55	50	
ITO/TiO_*X*_/TPPCOOHZn/Blend/MoO_x_/Ag		0.73	15.70	0.67	7.7	4.62	60	
ITO/ZnO/Blend/MoO_*x*_/Ag	4300	0.74	14.30	0.67	7.10	5.54	78	(53)
ITO/ZnO/Al/Blend/MoO_*x*_/Ag		0.75	15.20	0.70	8.00	6.80	85	
ITO/PEDOT:PSS/Blend/PDINO/Ag	1200	0.81	16.50	0.66	8.60	6.71	78	(55)
ITO/PEDOT:PSS/Blend/PDINO/PCB/Ag		0.82	17.60	0.70	9.9	9.70	98	
ITO/PEDOT:PSS/Blend/C60/Al	24	0.76	16.30	0.70	8.70	6.53	75	(56)
ITO/PEDOT:PSS/Blend/C60/Au		0.76	15.80	0.71	8.60	8.51	99	
ITO/Cu-Gr/PEDOT:PSS/Blend/C60/Au	720	0.80	16.3	0.65	8.5	8.33	98	(57)

a The estimated value from the degradation curve in the references.

Figure 5c presents the evaluation of the *J*–*V* characteristics in the dark of the representative degraded devices. The devices showed an increase in the leakage current with a 1 order of magnitude difference between the fresh and degraded cells. This greatly matched with the noticed reduction in the *J*_SC_ values of the degraded devices in Figure 5a. Moreover, it was surprising to find that D-30R-ZnO devices showed a bit lower leakage current after 8000 h, followed by an increase of 2-fold till the *T*_80_. This behavior might be attributed to the mentioned light soaking phenomenon of the inverted OPVs due to the diminishing of the photoinduced shunts, which enhances the *R*_Sh_,^46,47^ followed by a further increase, as illustrated in Figure 5b, confirming the increment of the *J*_SC_(48) during the degradation and then a decline till 12,000 h, as discussed previously.

Despite the information provided from the *J*–*V* characteristics that explained the effect of the ZnO modification on the performance parameters as well as the degradation behavior of the devices, an in-depth insight into the performance deterioration of the fresh and degraded cells cannot be obtained. Hence, IS characteristics were measured to gain a comprehensive understanding regarding the electric properties of the fresh and degraded devices upon the interfacial charge transfer and carrier recombination^58^ due to the modification of the ZnO film within the iF-OPVs. IS was used to measure the response of the device when an external alternating current was applied.^59^

**Figure 6:**
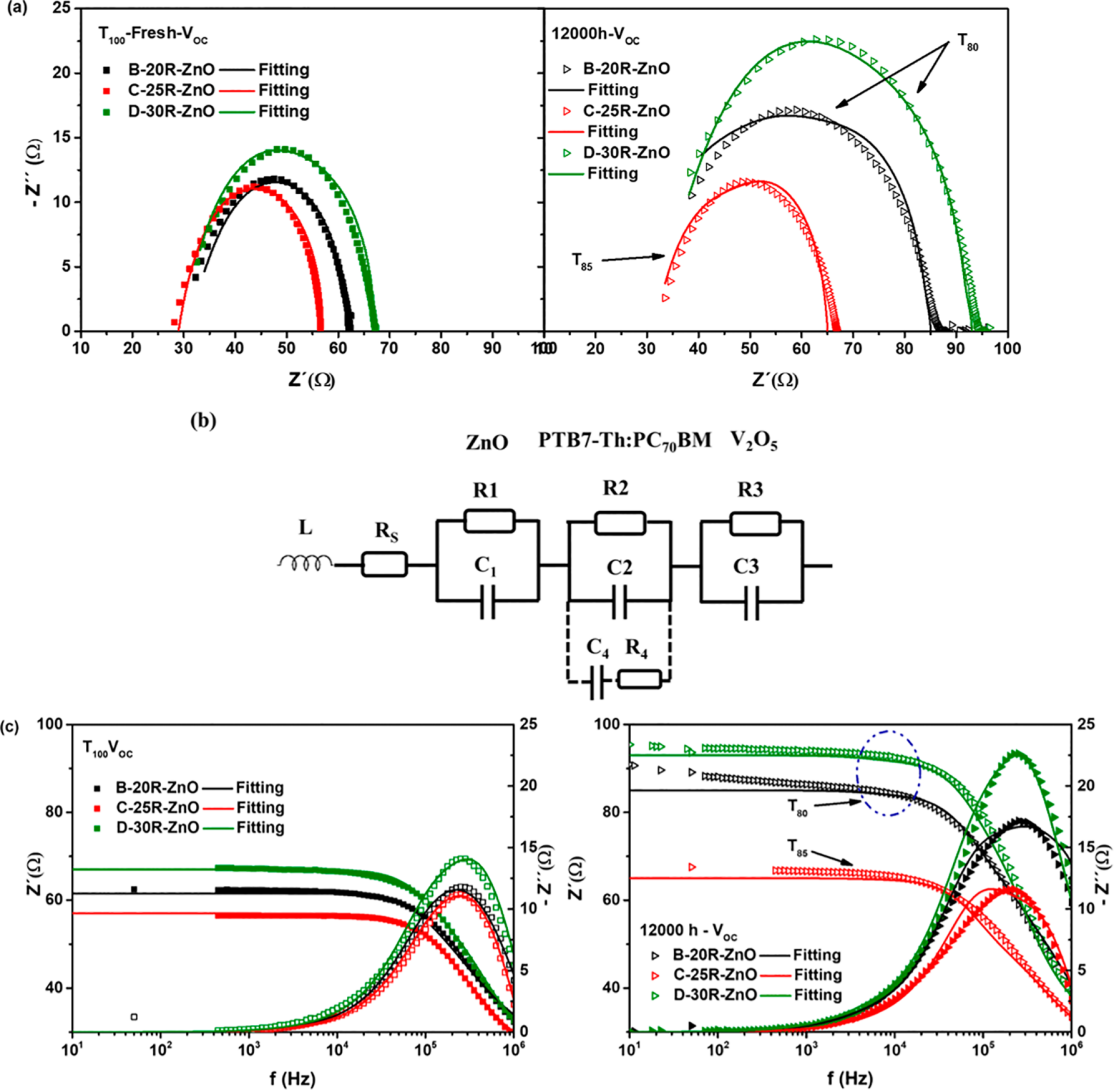
(a) Cole–Cole curves under AM 1.5G illumination at *V*_OC_ of the *T*_100-_fresh (left) and 12,000 h aged devices (right), symbols are presented for the experimental data and the fitting results in solid lines, applying (b) equivalent circuit using the Debye model. (c) Bode plot: experimental (symbols) and fitted (lines) values for the real part (left axis) and the imaginary part (right axis) of *T*_100_ for the fresh devices (left) and after 12,000 h for the degraded cells (right) at *V*_OC_. The fitted lines are obtained using the data demonstrated in Table S2.

Figure 6a shows the Cole–Cole plots for the *T*_100_ fresh and 12,000 h degraded devices under illumination at the open-circuit bias voltage with their corresponding Bode plots in Figure 6b, which reveals the efficient transfer at the active layer/electrode interfaces.^58^Figure 6a for the *T*_100_ fresh and 12,000 h degraded samples illustrates a typical semicircle curves with the Z′(Ω) real part of impedance as the *x*-axis and the Ź́(Ω) imaginary part as the y-axis. It can be noticed that the semicircle radii showed a variation in sizes from one device to another, which is basically correlated to the different charge injection or extraction that takes place within the device.^58,60,61^ Then, we observed that the C-based devices presented a smaller arc radius as well as lower impedance than the B- and D-based ones. The same behavior was obtained for the *T*_90_ degraded samples demonstrated in Figure S3. It is worth mentioning that the diameter of the arc radius increases as the aging time increases. However, the increase in the arc radius was noticeable for the B- and D-based *T*_80_-12,000 h degraded cells more than the C (*T*_85_-12,000 h)-based ones, verifying the more stable behavior of C devices. In addition, as Arredondo et al. suggested, the low-frequency impedance arc indicates charge accumulation, reflecting inadequate charge extracted by the contacts of the device.^50^ Accordingly, in Figure 6a, we can notice that the low-frequency arc was the smallest for the C-based devices as well as the arc size. This behavior reflects the effective charge extraction in the device leading to lower leakage current (Figures 2b and 5c), higher *V*_OC_, and, as a result, enhanced device performance and stability (Table 1, Figure 5b, and Table S1). On the other hand, this detected behavior confirms the lower performance of devices B and D (20R and 30R-ZnO—Table 1) as they possess a higher low-frequency arc, which might be related to the more pronounced charge accumulation effect that could be derived from the disorder of the blend morphology over the corresponding sprayed ZnO films.^30^ This consideration is correlated with the higher ZnO surface roughness obtained for B- and D-based films (Figure 3b,d) and higher leakage current and lower *V*_oc_ of the B- and D-based devices. Moreover, this behavior is in good agreement with a prior work.^26^ Interestingly, we exhibited a typical Cole–Cole plot attitude by applying similar measurements of *Z*′(Ω) versus Z″(Ω) at the maximum power point applied voltage (*V*_MPP_) and the short-circuit current for the *T*_100_-fresh and *T*_90_-12,000 h degraded samples, as shown in Figure S4.

As a consequence, to obtain an in-depth insight into the physical parameters of the fresh and degraded cells and to understand this behavior, we conducted an electrical equivalent circuit to fit the experimental *Z*′–*Z*″ data. The basic electrical circuit components used to fit the plots (solid lines) are presented in Figure 6b for the *T*_100_ fresh and 12,000 h degraded cells. The proper fitted parameters are listed in Table S2. This equivalent circuit possesses a distributed resistor (*R*), which refers to the electron transportation resistance of each layer within the device. Hence, *R*_1_, *R*_2_, and *R*_3_ components are for ZnO, the PTB7-Th:PC_70_BM active blend layer, and the V_2_O_5_ layer, respectively. In addition, *C* represents the geometrical capacitance values of each layer, exhibiting a parallel association of the resistor and capacitor for 3 *RC* resistive/capacitive components in series. Moreover, the *R*_S_ component represents the metallic wire series resistance as well as the resistance from the ohmic components such as ITO films and Ag electrodes,^8,58^ and L is the inductor used for proper fitting of the data at high frequency.^58^ It is interesting to notice that the model consists of an addition *R*_4_*C*_4_ unit included in series that is connected in parallel to the *R*_2_*C*_2_ unit of the PTB7-Th:PC_70_BM blend film following the Debye model,^8,62^ considering the presence of a single type of trap created in the corresponding film.^62^ Hence, the Debye model was the most proper one used to fit the obtained experimental data, clarifying the effect of the modified ZnO film on the performance and stability behavior of the iF-OPV devices.

From the fitting values listed in Table S2, we manifested that the fitting values of the capacitance of each layer matched greatly with the theoretical ones summarized in Table S3. This might reflect that at *V*_OC_, the impedance data were governed by the geometrical capacitances given by the metal insulator–metal (MIM) model, indicating a fully depleted layer behavior.^63,64^ Regarding the values of the resistance from the fitted data, we found out that for both *T*_100_ fresh and 12,000 h degraded devices, insignificant changes were observed for the *R*_S_ values from one device to another as well as with the aging time. This might reflect the negligible influence of the ZnO film modification on the resistance of the ITO film.

First, by detecting the effect of ZnO film modification on the *T*_100_ fresh devices, we found that the *R*_1_ values for the ZnO film of C-based devices (17.0 Ω) are less than that obtained in B and D devices (18.0 and 24.0 Ω, respectively). Furthermore, the values of *R*_2_ for the blend layer deposited over the sprayed ZnO films were 18.5, 10.0, and 13.0 Ω for the B-, C-, and D-based devices, respectively. Then, it can be clearly observed that the 25R ZnO film suppresses the *R*_2_ value for the blend, resulting in a better film quality that assists the charge transportation paths within the active blend layer, providing less impeded traps within the film.^58^ This behavior confirms the enhancement in the interface between the blend film and the ZnO interfacial film, which might be attributed to the homogeneity of the film morphology observed for the 25R-ZnO films, as discussed previously in the AFM analysis (Figure 3c). Regarding the V_2_O_5_ interfacial layer, we revealed no obvious effect upon the ZnO film modification within the fabricated devices. Accordingly, the total resistance values (*R*_Total_) evaluated for the cells (summarized in Table S2) were mainly controlled by the *R*_1_ value of the ZnO film. This showed the same trend behavior of *R*_1_ by representing the higher values for B- and D-based devices, confirming their low performance, and the smallest *R*_Total_ and *Z*′ values along with the smallest arc radius for device C, confirming the enhanced iF-OPV performance. We can notice that the mentioned resistance behavior obtained for the devices was verified by the Bode plot in Figure 6c—*T*_100_. Interestingly, these observed results greatly matched with the lowest value of *R*_S_, highest value of the FF, and in turn the champion PCE of the C-based devices from the *J*–*V* characteristics under a similar illumination condition (Figure 2 and Table 1).

Second, regarding the fitted resistance values of 12,000 h degraded devices listed in Table S2, we noticed that *R*_1_ values related to the ZnO film remained lower for C-based devices (16.0 Ω) than for B and D ones (17.0 and 35.0 Ω). However, B- and C-based devices succeeded in keeping their similar initial resistance value (*T*_100_) after 12,000 h, but the degraded D-based devices showed a pronounced enhancement of their *R*_1_ value by 45% compared to that of the fresh ones (Figure S5 and Table S2). Furthermore, the observed fitted *R*_2_ values (of the blend) for the 12,000 h devices were increased by 70, 30, and 43% of their initial values (*T*_100_) for the B-, C-, and D-based cells, respectively. Otherwise, the *R*_3_ values of the V_2_O_5_ film were increased by almost similar percentages of ≈40% for the entire degraded cells (Figure S5). Since the *R*_S_ values (regarding the ITO) were almost similar for the fresh and degraded devices, we can assume that the traps created during the time of the degradation mainly affect the blend interface with the ZnO ETL and the V_2_O_5_ hole transport layer. Accordingly, we can disclose that the degraded devices were controlled by the *R*_2_ value of the blend as the *R*_Total_ obeyed a similar trend by showing the lowest value for the C-based devices (65.0 Ω) and the highest for the D-based devices (93.0 Ω), as listed in Table S2. This might confirm the lowest Z′ values measured and their lowest arc size (Figure 6a—12,000 h) of the C devices, reflecting the highest stability obtained for the corresponding devices (Figure 5). This variation of the resistance behavior obtained was clarified by the Bode plots in (Figure 6c—12,000 h). This can be described by the fact that less pronounced defects were created within the blend layer of the C devices, as ascribed by the value of resistance evaluated from the Debye model.^62^ Therefore, Figure S5 demonstrates that C-based devices show the highest consistency of the resistance values in each layer till 12,000 h. In addition, this observation confirmed the *J*–*V* characteristics for the stability study, which clarifies that after 12,000 h, C devices retained 85% of their performance while the other iF-OPVs reached 80% of their performance. Based on these results, it is apparent that the properties of the electron transporting interfacial layer (ZnO film modification) can have a significant impact on the stability of the OPV devices.

It was surprising to note that all the degraded devices prepared using the intermittent spray pyrolysis technique showed a single arc of impedance, which is in agreement with our previous work.^8^ However, in that previous work, the Cole–Cole plots showed an extra arc for the degraded samples prepared using the spin coating technique, which verifies the efficient degradation inhibition provided by the modification of the ZnO interfacial layer upon the variation of the deposition techniques. Furthermore, the comparison between the previous and current samples prepared using the intermittent spray pyrolysis technique indicates that the concentration of the ZnO precursor solution and the deposition parameters play a crucial role in enhancing the device performance and stability.

A further procedure to study the behavior of the recombination mechanisms through the IS technique is the capacitance-frequency (*C*_f_) measurements to evaluate the trap DOS. It is known that the main source of traps in the OPVs is the disorder.^65^ Therefore, this characterization was carried out for OPVs in many reported research studies to investigate the change in trap emission along with the imperfection and energy disorder-exhibiting tail states inside the cells.^8,58,65,66^ Accordingly, at a given energy level, *E*_ω_, we can calculate the traps DOS by varying the capacitance with respect to the frequency of the device. This corresponds to the charge release by shallow traps in the band gap close to the Fermi energy level, as given by eq 1(65,66)
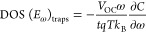
1where *V*_OC_ is the open-circuit voltage from the *J*–*V* characteristics (Table 1) under illumination conditions, *C* is the measured capacitance, ω is the angular frequency, *k*_B_ is the Boltzmann constant, *t* is the thickness of the layer, *q* is the electron charge, and *T* is the room temperature (around 300 K).

To evaluate the relation of the dependency of energy on the trap DOS, we applied the following equation
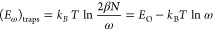
2where *N* is the effective DOS and β is the cross-section.^67^ By assuming that 2βN does not depend on of the value of the frequency, the value variation is corelated to the shift in the DOS values on the energy scale (*E*_O_).^66^

**Figure 7:**
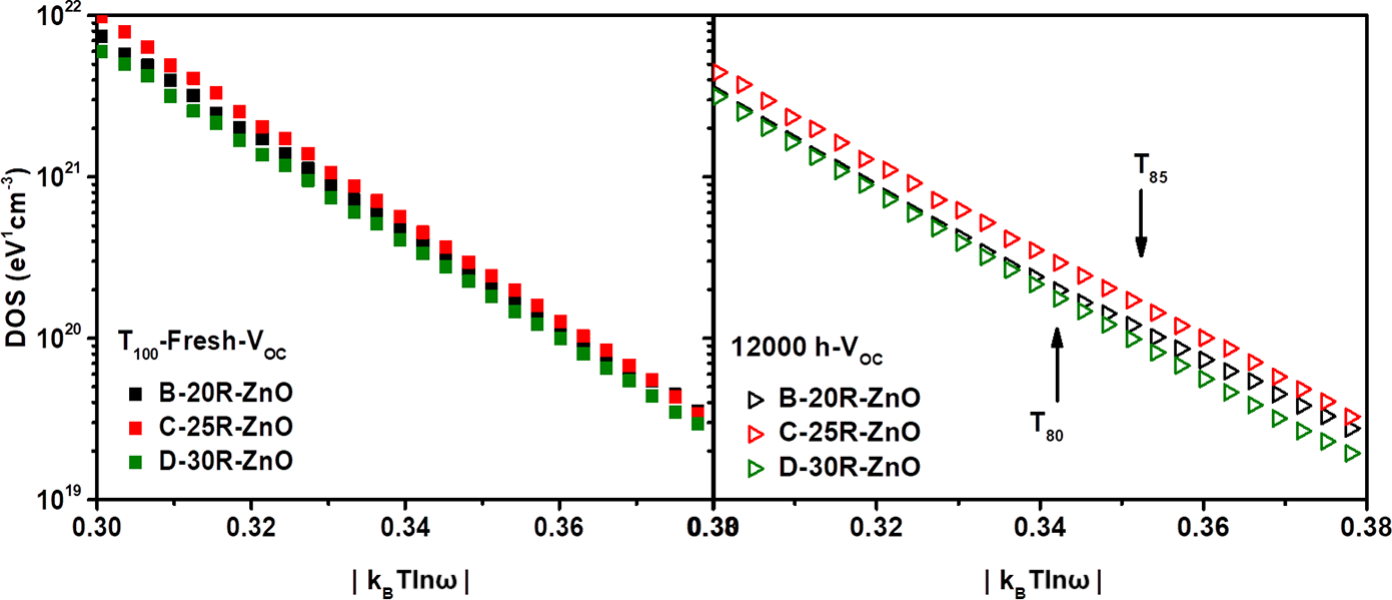
DOS vs |*k*_B_*T* ln ω| under AM 1.5G illumination at *V*_OC_ of the *T*_100_ fresh devices (left) and the 12,000 h degraded (right) iF-OPVs.

Figure 7 displays the calculated trap DOS plotted as a function of energy for *T*_100_ and 12,000 h devices. We noticed that all samples exhibited the same carrier response and trap activation energy, showing a single exponential trap distribution with almost the same slope values.^68^ For the *T*_100_ fresh samples, we did not observe a significant change in the DOS value for all devices. However, C-based devices presented slightly higher DOS values than the others. This behavior is greatly consistent with the *E*_U_ results discussed previously in Figure 4c and clarifies the impedance behavior as well in Figure 6a, along with explaining the higher FF and *V*_OC_ obtained for C-based devices in Figure 2a and Table 1. Thus, it might be correlated to the enhanced 25R-ZnO film quality, as discussed previously in Figure 3c.

Then, for the 12,000 h degraded devices, we can observe a decrease in the DOS values for a given frequency. This behavior can be explained by eq 2 as a shift in the *E*_O_ value upon increasing the βN value. Hence, the degraded devices showed lower energy, representing higher interfacial-induced defects in the devices, than the fresh ones as an intrinsic source of traps.^8,65,67,69^ Moreover, it was interesting to find out that the DOS value for the 12,000 h degraded C-based devices was higher than that of B- and D-based devices (Figure 7-12,000 h). The energy shifting values (*X*) calculations are clarified in the Supporting Information. By performing a comparative analysis for the values of the energy shift of the 12,000 h degraded devices in Figure S6, we obtained that the B- and D-based devices exhibited a higher shifting value than the C-based one. This indicates that less trapped sites are located in device C, verifying their superior stable behavior toward the degradation. This detected behavior interestingly agreed with the obtained 12,000 h *J*–*V* characteristics in Figure 5 as well as the impedance behavior given in Figure 6a. Furthermore, the opposite behavior of D-based devices confirms the increase of the interface DOS values for this sample, which is correlated with its lower *V*_OC_, PCE, and the diminished stability behavior. Interestingly, the same attitude was distinguished for the *T*_90_ degraded devices presented in Figure S7.

Accordingly, the observed data showed that modifying the ZnO film via spraying 25R using the intermittent spray pyrolysis approach enhanced the ZnO film quality as well as that of the attached active blend film. Therefore, it was interesting to mention that this modification assists the stability of the fabricated devices (C-based devices) more than that for the other cells. In addition, comparing with the previously reported work,^8^ we can clearly notice the significant stability enhancement obtained by the modified 25R-ZnO film sprayed with a low concentration of the ZnO precursor solution (reached *T*_85_ after 12,000 h) compared to that of the ZnO film spin-coated and the ZnO film sprayed using a high concentration of the ZnO precursor solution (reached *T*_85_ after 5000 h). This might be attributed to the better ZnO film quality obtained using the 25R fabrication of low ZnO precursor solution, which provides less defect sites for the over-coated blend layer, which in turn plays an important role in enhancing the device performance and stability.^68^

## 4 Conclusions

In summary, the importance of the surface roughness of the ZnO layer (ETL) in identifying the PV performance parameters of the iF-OPVs was detected by using PTB7-Th:PC_70_BM as the photoactive layer. We demonstrated that the careful tuning of the microstructure features, morphology, and properties of the sprayed ZnO film via a low concentration precursor solution depends sensitively on the chosen number of spraying running cycles using the intermittent spray pyrolysis approach. Hence, the optimized morphology of the sprayed ZnO (with the lowest roughness and full surface coverage) was achieved through the 25R condition, yielding the best performance of a 9.86% PCE along with an enhanced average *V*_OC_ (0.80 V) and FF (0.67) for the C-based device. Furthermore, we tested their stability behavior, which demonstrated a pioneer record with respect to device performance, maintaining 85% of the starting efficiency even after 16.7 months of storage without encapsulation inside a nitrogen glovebox. The difference in the device performance and stability appears to originate from the different ZnO surface morphologies obtained that control the presence of defects at the surface and their subsequent adjacent organic active layer blend. As a sequence, the surface roughness determines the effective interfacial region between the active layer and the ZnO layer and thus the density of trap sites at the interface that was investigated by the IS measurements for the fresh and degraded iF-OPVs. Therefore, the proposed electrical equivalent circuit module fitting the experimental data of the IS allowed us to recognize the impact of each interlayer on the device performance and the correlated stability behavior. Accordingly, we showed that the remarkable stability enhancement behavior of the C-based devices correlates with the marginal interface DOS values for this sample among the others. Finally, it is worth mentioning that the obtained high efficiency and excellent stability of the fabricated inverted OPVs using the spray pyrolysis technique could facilitate their scale up to the industrial production approach.

## Supporting Information Available

Performance parameter details of the freshly and aged fabricated iPSCs, explanation of the behavior of the fresh and degraded devices using the *J*–*V* curves obtained under illumination, IS, and DOS, and equivalent electrical circuit used for the fitting of the experimental data of Nyquist plots (PDF)

## Abbreviations

iF-OPVs: inverted fullerene organic solar cells
SP: spray pyrolysis
R: spraying running cycles
*V*_OC_: open-circuit voltage
FF: fill factor
*R*_S_: series resistance
*R*_SH_: shunt resistance
*J*_SC_: current density
PCE: power conversion efficiency
*J*–*V*: current density–voltage
EQE: external quantum efficiency
*E*_U_: Urbach energy
IS: impedance spectroscopy
DOS: density of states
