# Supplementary material for: Significant Stability Improvement of Fullerene Organic Photovoltaics via ZnO Film Modification through the Intermittent Spray Pyrolysis Technique
Source: ACS Appl Energy Mater. 2022 Mar 29;5(4):4390–403. doi: 10.1021/acsaem.1c03994 (PMC9045677; doi:10.1021/acsaem.1c03994)
Supplement: Supplementary file 1 — ae1c03994_si_001.pdf [file ae1c03994_si_001.pdf]

Supporting Information

Significant Stability Improvement of Fullerene Organic Photovoltaic via ZnO Film Modification through the Intermittent Spray Pyrolysis Technique

Enas Moustafa, [a] *Lluis F. Marsal,* [a]* *and Josep Pallarès* [a]*

[a] Department of Electrical Electronic Engineering and Automatic, Universitat Rovira i Virgili, 43007, Tarragona, Spain

*Email: lluis.marsal@urv.cat and josep.pallares@urv.cat

KEYWORDS: stability of fullerene organic photovoltaics, ZnO electron transporting layer, thin film deposition techniques, intermittent spray pyrolysis, degradation mechanisms in organic photovoltaics.

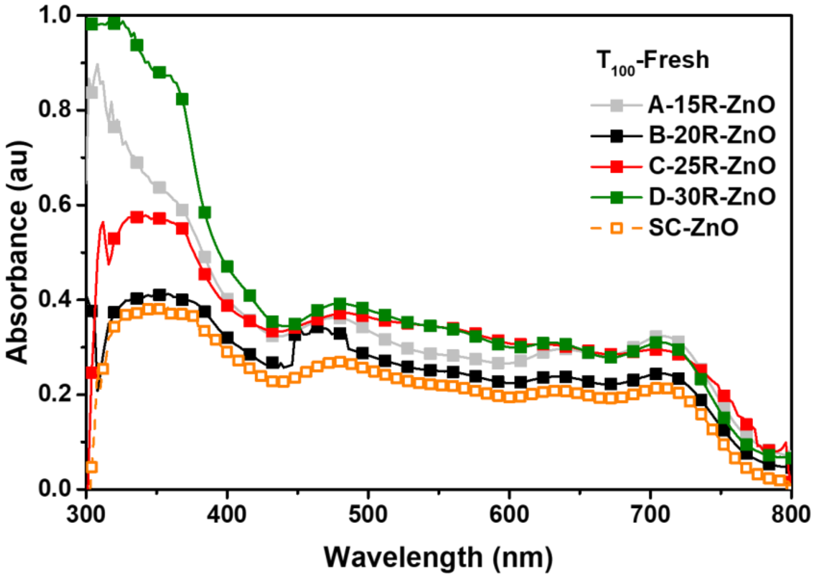

Figure S1. UV-Vis optical absorbance characteristics of the various ZnO/active blend films, where ZnO films deposited by intermittent spray pyrolysis (15R, 20R, 25R and 30R) along with the ZnO film coated by spin coating technique reported in our previous work[1].

Table S1. Photovoltaic performance parameters statistics of the degraded inverted fullerene OPVs that presented from average of at least 9 devices.

AGING TIME (h)		V_{OC} (V)	J_{SC} (mA/cm²)	FF	PCE (%)	PCE_{MAX} (%)	NORMALIZED PCE (%)	R_S (Ω cm²)	R_{SH} (Ω cm²)
**B-20R**									
**T100**	**0**	0.79 ± 0.01	18.26±0.12	0.66 ± 0.01	9.71 ± 0.09	9.80	100	2.83 ± 0.32	765 ± 17
**≈T95**	**400**	0.79 ± 0.01	17.98 ± 0.21	0.65 ±0.01	9.26 ± 0.10	9.36	95	2.92 ± 0.44	613 ± 21
	**6000**	0.79 ± 0.01	17.96 ± 0.09	0.63 ± 0.01	9.01 ± 0.20	9.21	93	2.81 ± 0.17	676±25
**≈T90**	**8000**	0.79 ± 0.01	18.03 ± 0.49	0.58 ± 0.02	9.00 ± 0.10	9.10	90	3.26 ± 0.31	394 ± 19
**≈T85**	**9000**	0.78 ± 0.01	17.98 ± 0.26	0.57 ± 0.02	8.11 ± 0.14	8.25	84	4.40 ±0.92	357 ± 52
**≈T80**	**12000**	0.78 ± 0.01	17.00 ± 0.91	0.56 ± 0.02	7.72 ± 0.15	7.87	80	4.50 ± 0.66	352 ± 12
**C-25R**									
**T100**	**0**	0.80 ± 0.01	18.42 ± 0.19	0.67± 0.04	9.80 ± 0.06	9.86	100	2.31 ± 0.11	774 ± 24
	**400**	0.80 ± 0.01	19.46 ± 0.73	0.61 ± 0.06	9.62 ± 0.05	9.67	98	2.39 ± 0.10	520 ± 17
**≈T95**	**6000**	0.79 ± 0.01	19.23 ± 0.61	0.62 ± 0.08	9.32 ± 0.05	9.37	95	2.46 ± 0.03	416 ± 52
	**8000**	0.79 ± 0.01	17.44 ± 0.33	0.66 ±0.02	9.18 ± 0.10	9.28	94	2.53 ± 0.22	757 ± 11
**≈T90**	**9000**	0.79 ± 0.01	18.39 ± 0.29	0.60 ± 0.07	8.66 ± 0.13	8.79	89	2.73 ± 0.16	387 ± 72
**≈T85**	**12000**	0.79 ± 0.01	16.51 ± 0.25	0.64 ± 0.04	8.37 ±0.03	8.40	85	2.91 ± 0.09	698 ± 13
**D-30R**									
**T100**	**0**	0.78 ± 0.02	18.88 ± 0.31	0.63 ± 0.03	9.46 ± 0.14	9.60	100	3.07 ± 0.43	381 ± 19
	**400**	0.78 ± 0.02	19.03 ± 0.61	0.62 ± 0.02	9.23 ± 0.26	9.49	99	3.09 ± 0.16	480 ± 09
**≈T95**	**6000**	0.78 ± 0.02	19.17 ± 0.22	0.60 ± 0.01	9.11 ± 0.20	9.31	97	3.36 ± 0.48	359 ± 12
**≈T90**	**8000**	0.78 ± 0.02	17.44 ± 0.19	0.61 ± 0.03	8.46 ± 0.04	8.51	87	3.40 ± 0.17	486 ± 07
**≈T85**	**9000**	0.78 ± 0.02	18.60 ± 0.21	0.58 ± 0.02	8.03 ± 0.10	8.13	85	4.61 ± 0.11	307 ± 16
**≈T80**	**12000**	0.77 ± 0.01	17.63 ± 0.15	0.57 ± 0.01	7.02 ± 0.14	7.16	76	4.80 ± 0.29	268 ± 13

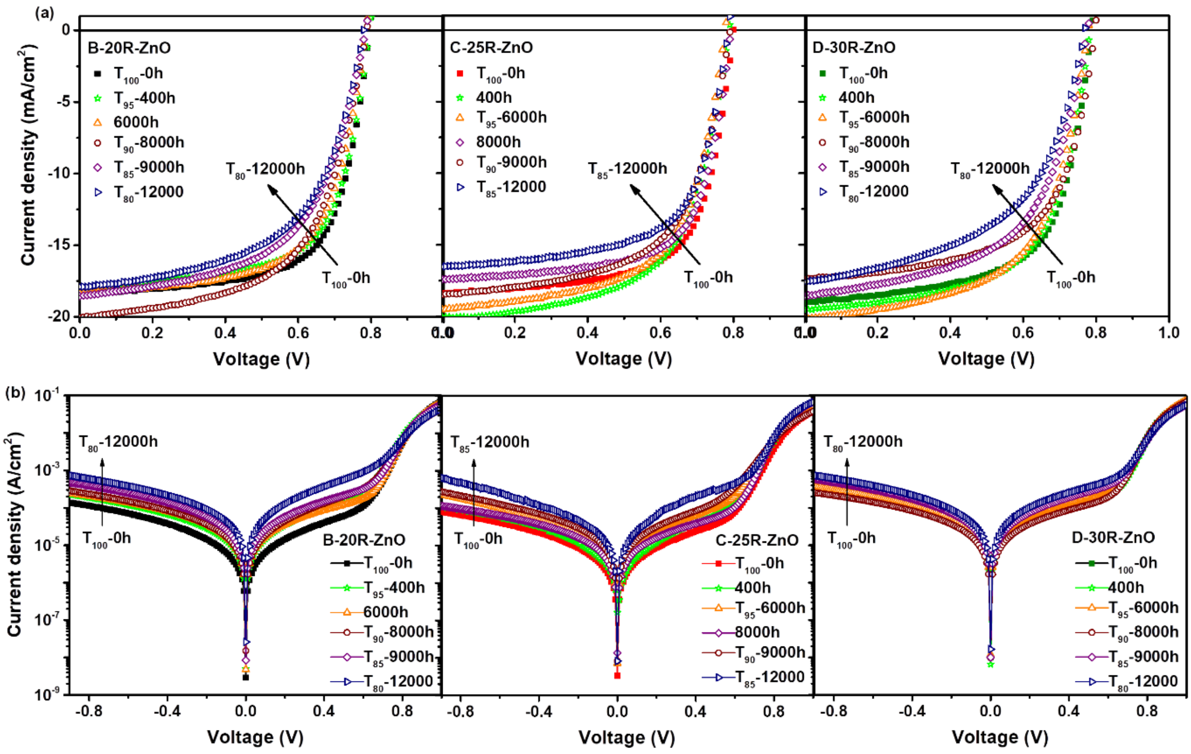

Figure S2. (a) Current density- voltage (J-V) characteristic curves under AM 1.5 G illumination, (b) at dark of the degraded iF-OPVs with respect to the aging time (12000 h)-devices stability study

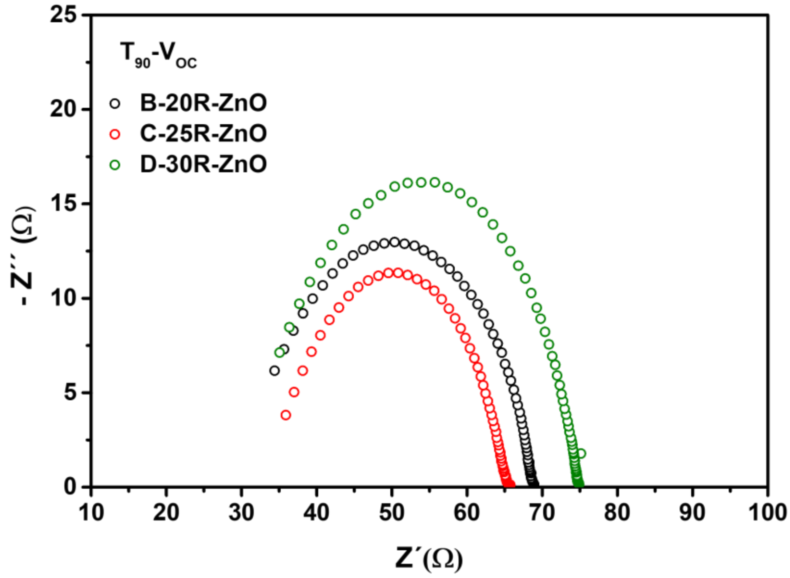

Figure S3. Cole-cole plots at V_{OC} under AM 1.5G illumination of the T_{90} aged iF-OPVs.

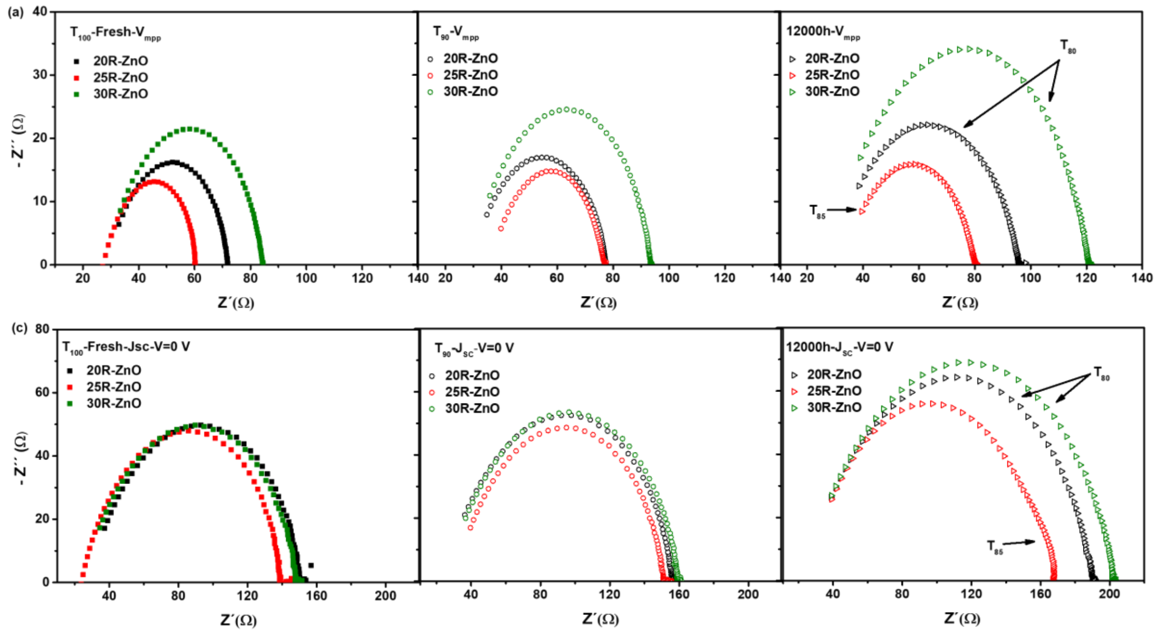

Figure S4. Cole-cole plots under AM 1.5G illumination of the T_{100}-fresh, T_{90} and 12000 h degraded devices at (a) maximum power point voltage, V_{mpp} (b) Short circuit current condition at voltage = 0 V.

Table S2. The fitted parameters of the equivalent circuit using Debye model at V_{OC} for the fabricated T_{100}-fresh and 12000 h degraded devices. The parameter $\tau = R_4 \times C_4$, where R_4, C_4 parameters were demonstrated in the equivalent circuit in the manuscript in **Figure 6b.**

**Physical Parameters of the T_{100}-Fresh iF-OPVs**	**Device B (20R-ZnO)**			**Device C (25R-ZnO)**		
**ZnO**	R_S = 14.0 Ω<br>L = 1.20 µH	R_1= 18.0 Ω	C_1= 26.2 nF	R_S = 17.0 Ω<br>L = 1.20 µH	R_1= 17.0 Ω	C_1= 19.1 nF
**PTB7-Th-$PC_{70}BM$**		R_2= 18.5 Ω<br>τ = 30.0 µs	C_2= 2.8 nF<br>C_4= 30.0 nF		R_2= 10.0 Ω<br>τ = 30.0 µs	C_2= 2.8 nF<br>C_4= 50.0 nF
**V_2O_5**		R_3= 11.0 Ω	C_3= 99.0 nF		R_3= 12.0 Ω	C_3= 99.0 nF
		R_{Total} = 61.5 Ω			R_{Total} = 56.0 Ω	
	**Device D (30R-ZnO)**					
**ZnO**	R_S = 16.0 Ω<br>L = 1.20 µH	R_1= 24.0 Ω	C_1= 13.7 nF			
**PTB7-Th-$PC_{70}BM$**		R_2= 13.0 Ω<br>τ = 30.0 µs	C_2= 2.8 nF<br>C_4= 100.0 nF			
**V_2O_5**		R_3= 14.0 Ω	C_3= 99.0 nF			
		R_{Total} = 67.0 Ω				
**Physical Parameters of the T_{80}- Degraded iF-OPVs**	**Device B (20R-ZnO)**			**Device C (25R-ZnO)**		
**ZnO**	R_S = 14.0 Ω<br>L = 1.20 µH	R_1= 17.0 Ω	C_1= 26.6 nF	R_S = 17.0 Ω<br>L = 1.20 µH	R_1= 16.0 Ω	C_1= 19.1 nF
**PTB7-Th-$PC_{70}BM$**		R_2= 34.0 Ω<br>τ = 30.0 µs	C_2= 2.8 nF<br>C_4= 30.0 nF		R_2= 14.0 Ω<br>τ = 30.0 µs	C_2= 2.8 nF<br>C_4= 50.0 nF
**V_2O_5**		R_3= 20.0 Ω	C_3= 99.0 nF		R_3= 18.0 Ω	C_3= 99.0nF
		R_{Total} = 85.0 Ω			R_{Total} = 65.0 Ω	
	**Device D (30R-ZnO)**					
**ZnO**	R_S = 17.0 Ω<br>L = 1.20 µH	R_1= 35.0 Ω	C_1= 13.7 nF			
**PTB7-Th-$PC_{70}BM$**		R_2= 21.0 Ω<br>τ = 30.0 µs	C_2= 2.8 nF<br>C_4= 100.0 nF			
**V_2O_5**		R_3= 20.0 Ω	C_3= 99.0 nF			
		R_{Total} = 93.0 Ω				

Table S3. Dielectric constants and calculated capacitances for each layer of the fabricated iF-OPVs.

Layers	ε_{Layer}	Capacitance (nF)	Thickness (nm) d_{Layer}
**V_2O_5**	5[2]	99	4
**Blend-PTB7-Th-PC$_{70}$BM**	3.5[3–5]	2.8	100
**B-20R-ZnO**	6[1,6]	26.6	18
**C-25R-ZnO**	6	19.1	25
**D-30R-ZnO**	6	13.7	35

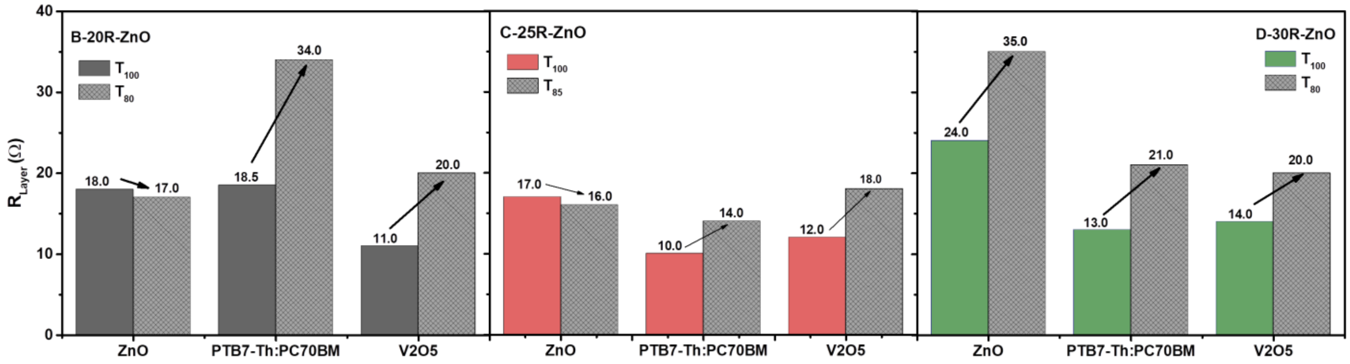

Figure S5. The resistance values of each layer for the T_{100}-fresh and T_{80}-degraded B (20R-ZnO), C (25R-ZnO) and D (30R-ZnO) based iF-OPVs. The values were extracted from the Debye model for the T_{100} fresh and 12000 h degraded devices.

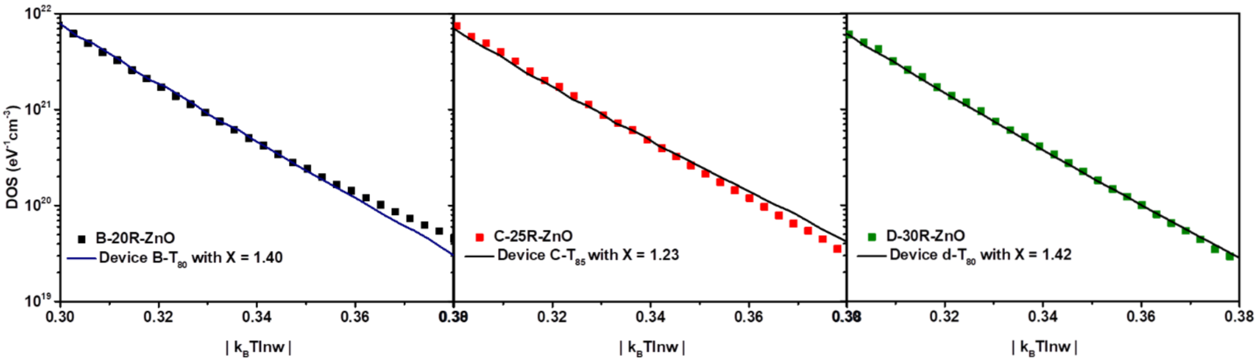

Figure S6. Density of state (DOS) as a function of energy at V_{OC} of the T_{100} fresh (symbols) and the 12000 h degraded devices (line with shifting value of X value).

The shifting of the 12000 h degraded devices shown in Figure 7 in the manuscript, defined by X, has been calculated as follows[1]:

Using Equation (2) in the manuscript, namely $(E_\omega)_{traps} = k_B \, T \ln\frac{2\beta N}{\omega} = E_o - k_B T \ln\omega$, we get the energy dependence on the frequency for the T_{100} fresh and 12000 h degraded cells as

$E_{F\,CN} = k_BT \ln\frac{\alpha_F}{\omega}$ and $E_{0D} = k_BT \ln\frac{\alpha_D}{\omega}$, respectively, where the parameter α = 2βN has been defined.

So, the energy's difference between the degraded and the fresh devices can be ascribed as

$$E_D - E_F = E_{O\,(D)} - E_{O\,(F)} = k_BT \ln\alpha_D - k_BT \ln\alpha_F = k_B\, T \ln\frac{\alpha_D}{\alpha_F}$$

Finally, the shifting value due to degradation can be obtained as

$$X = \exp\left(\frac{E_D - E_F}{k_BT}\right) = \frac{\alpha_D}{\alpha_F} = \frac{\beta_D.N_D}{\beta_F.N_F}$$

where β is the cross-section and N is the effective density of states.

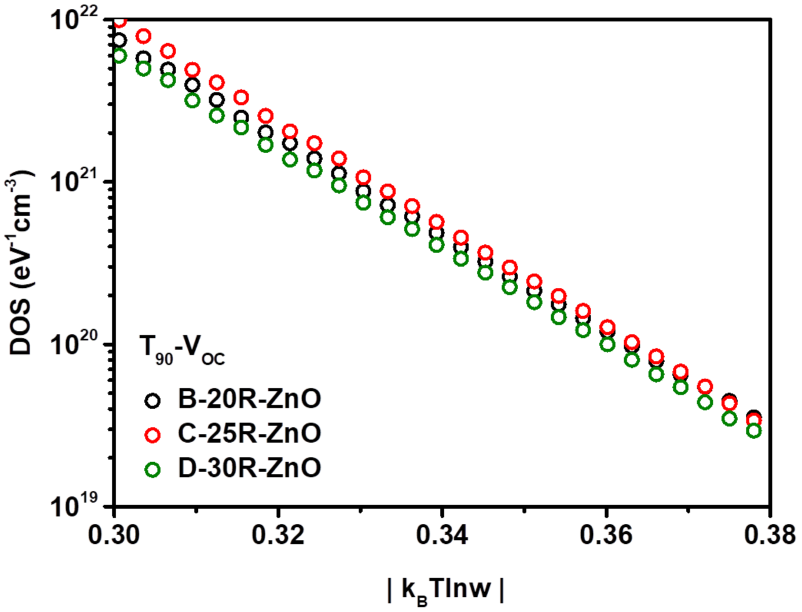

Figure S7. DOS as function of $|k_BT\ln\omega|$ at V_{OC} under AM 1.5G illumination of the T_{90} degraded iF-OPVs.

References

(1) Moustafa, E.; Sánchez, J. G.; Marsal, L. F.; Pallarès, J. Stability Enhancement of High-Performance Inverted Polymer Solar Cells Using ZnO Electron Interfacial Layer Deposited by Intermittent Spray Pyrolysis Approach. *ACS Applied Energy Materials* **2021**, *4* (4), 4099–4111.

(2) Osorio, E.; Sánchez, J. G.; Acquaroli, L. N.; Pacio, M.; Ferré-Borrull, J.; Pallarès, J.; Marsal, L. F. Degradation Analysis of Encapsulated and Nonencapsulated TiO_2/PTB7:$PC_{70}BM$/V_2O_5 Solar Cells under Ambient Conditions via Impedance Spectroscopy. *ACS Omega* **2017**, *2* (7), 3091–3097.

(3) Torabi, S.; Jahani, F.; Van Severen, I.; Kanimozhi, C.; Patil, S.; Havenith, R. W. A.; Chiechi, R. C.; Lutsen, L.; Vanderzande, D. J. M.; Cleij, T. J.; Hummelen, J. C.; Koster, L. J. A. Strategy for Enhancing the Dielectric Constant of Organic Semiconductors without Sacrificing Charge Carrier Mobility and Solubility. *Advanced Functional Materials* **2015**, *25* (1), 150–157.

(4) Garcia-Belmonte, G.; Munar, A.; Barea, E. M.; Bisquert, J.; Ugarte, I.; Pacios, R. Charge Carrier Mobility and Lifetime of Organic Bulk Heterojunctions Analyzed by Impedance Spectroscopy. *Organic Electronics* **2008**, *9* (5), 847–851.

(5) Sánchez, J. G.; Balderrama, V. S.; Estrada, M.; Osorio, E.; Ferré-Borrull, J.; Marsal, L. F.; Pallarès, J. Stability Study of High Efficiency Polymer Solar Cells Using TiO_x as Electron Transport Layer. *Solar Energy* **2017**, *150*, 147–155.

(6) Ondo-Ndong, R.; Essone-Obame, H.; Moussambi, Z. H.; Koumba, N. Capacitive Properties of Zinc Oxide Thin Films by Radiofrequency Magnetron Sputtering. *Journal of Theoretical and Applied Physics* **2018**, *12* (4), 309–317.
